# Pro-inflammatory Cytokines, Ferroptosis, and Cancer

**DOI:** 10.32607/actanaturae.27547

**Published:** 2025

**Authors:** A. A. Vartanian, V. S. Kosorukov

**Affiliations:** N.N. Blokhin National Medical Research Center of Oncology of the Ministry of Health of Russia, Moscow, 115478 Russian Federation

**Keywords:** ferroptosis, intratumoral immunosuppression, pro-inflamatory cytokines, cancer

## Abstract

Ferroptosis, iron-dependent regulated cell death, is induced by the polyunsaturated fatty acid peroxidation of membrane phospholipids and is controlled by glutathione peroxidase 4. In recent years, convincing evidence has emerged, demonstrating a close relationship between chemo-, radio-, immuno-, and targeted therapy resistance and ferroptosis resistance. In this review, we discuss the basic principles of ferroptosis in cancer. Considerable attention is paid to the formation of an immunosuppressive tumor microenvironment. The main focus is centered on the involvement of the excessive, chronic production of pro-inflammatory cytokines in ferroptosis resistance development in tumors.

## INTRODUCTION

The concept holding that changes in the tumor cell genome contribute little to the progression of malignancy is now generally accepted [1]. The behavior of a tumor cell – survival, proliferation, and transition to the metastatic growth phase – is controlled by its microenvironment (extracellular matrix, neighboring non-transformed cells, immune system cells, blood). The tumor microenvironment also regulates resistance to therapy [2].

Ferroptosis, an iron-dependent form of cell death, was first reported in 2012 [3]. In ferroptosis, excess Fe^2+^ ions, which are not bound to proteins, trigger the Fenton reaction:

Fe^2+^ + H_2_O_2_ → Fe^3+^ + OH– + •OH.

The Fenton reaction-generated hydroxyl radical is highly reactive and capable of oxidizing virtually any cellular component. The central mediator in ferroptosis is the accumulation of polyunsaturated fatty acid peroxidation products in the cell [4]. It should be noted that ferroptosis activation does not require the processing of cell death effectors, such as caspases or gasdermins: ferroptosis is an energetically less expensive cell death process. Data on the sensitivity of chemotherapy-, radiotherapy-, and targeted therapy-resistant tumor cells to ferroptosis have considerably heightened interest in the phenomenon [5].

The transition of a tumor to the aggressive growth phase occurs when control by the immune system is incomplete. Dysfunction of both tumor-infiltrating and circulating T cells has been reported (see review [6]). Also, the tumor induces significant changes in macrophages: polarization of macrophages to the M2 phenotype is observed [7]. Tumor cell secreted cytokines recruit Tregs to the tumor. The number of Tregs in the tumor increases at all stages of the disease; they suppress the proliferation and functional activity of CD4^+^ and CD8^+^ T cells [8]. MDSCs also play an important role in suppressing the immune response [9]. Reprogramming of antitumor immunity, which results in the stimulation of primary tumor growth by immunocompetent cells of the microenvironment, as well as instability of the tumor cell genome, is now considered a factor that confers protection from the immune system to the tumor and facilitates its progression.

This review briefly discusses the features of iron metabolism in cancers, the main characteristics of ferroptosis, and the involvement of the chronic production of pro-inflammatory cytokines in tumor progression. Particular attention is paid to the development of tumor resistance to ferroptosis.

## IRON IN THE TUMOR MICROENVIRONMENT

Until recently, tumor progression was studied in terms of the dependence of tumor cell survival on important metabolites, such as glucose and glutamine. But today, there is probably no doubt that the iron in the tumor microenvironment is also an important component of tumor cell survival. This is now quite obvious, since iron performs a number of metabolically important functions in the cell, delivering oxygen to tissues (heme) or acting as a cofactor for several enzymes; e.g., the ribonucleotide reductase that is involved in the biosynthesis of DNA or Krebs cycle enzymes [10]. The proliferation of tumor cells can be controlled through the regulation of iron reserves in the tumor. Also, iron is used not only by tumor cells, but also by the cells of the tumor microenvironment.

Cells take up iron mainly through receptor-mediated endocytosis of the Tf/iron complex. Binding of the Tf/iron complex to its receptor, TfR1 (CD71), results in ingestion of the Tf/iron/CD71 complex into the cell. In the cell, iron dissociates from the complex in endosomes and is incorporated into iron-dependent proteins, whereas the receptor and Tf return to the cell surface [11]. Non-protein-bound iron is stored in the cell as a complex with ferritin [12]. Free iron, not bound to proteins and ferritin, is excreted from the cell via a membrane-bound protein, FPN, or becomes part of the labile iron pool [13]. Iron levels in the body are regulated by the peptide hormone hepcidin. In response to increased iron concentrations in the blood, hepatocytes activate hepcidin expression and secretion into the blood stream. Binding of hepcidin to FPN promotes ingestion of both proteins into the cell and their degradation in lysosomes. This blocks the release of iron from depot cells, which reduces the plasma iron level. In the case of iron deficiency, hepcidin transcription is suppressed [14]. Iron binding by proteins not only maintains cell viability, but also protects cells from the highly reactive hydroxyl radical generated in the Fenton reaction. It should be noted that our body has developed fairly strict control mechanisms for self-protection against changes in the iron metabolism. This means that our cells fail to control iron reserves only in extreme cases; in particular, cancers.

Tumor cells require significantly more iron to maintain a high proliferation index; so, TfR1 expression in tumor cells is increased to compensate for iron deficiency. Tumor cells also accumulate ferritin, depositing iron. The expression of FPN that exports iron from the cell also changes. A decreased FPN expression is observed in most malignancies (see review [15]). These successive processes lead to decreased blood iron levels and, thereby, to extremely low hemoglobin levels in cancer patients. Intravenous iron infusions, which normalize hemoglobin levels in anemia, usually do not increase hemoglobin levels in cancer patients. An autopsy reveals that most of the iron is deposited in the liver [16]. Reprogramming of the iron metabolism, which promotes iron accumulation in the tumor cell, is typical of all tumor types.

Today, an aggressive course of the disease is also believed to be associated with mutations in the tumor cell. It is important to note that mutations in many oncogenes (*c-myc*, *KRAS*, *BRAF*, *PI3K*) and deletion in *PTEN *promote an increase in iron levels inside the cell, and that mutations in tumor suppressor genes (*p53*) shrink the labile iron pool [17, 18]. An aggressive course of the tumor process is also associated with the accumulation of iron-secreting M2 macrophages in the tumor. Therefore, to increase the uptake of iron and reduce its loss, it is not enough to initiate changes in the expression of the proteins that control iron levels in tumor cells. Cross-talk between tumor cells and microenvironment cells is also nessesary. It should also be noted that the tumor microenvironment constantly changes.

## FERROPTOSIS IS IRON-DEPENDENT REGULATED CELL DEATH

In 2018, the Nomenclature Committee on Cell Death officially defined ferroptosis as regulated cell death caused by abnormal oxidation of the polyunsaturated fatty acids of membrane phospholipids and controlled by GPX4.

This form of cell death is associated with hydroxyl radical generation in the Fenton reaction. In ferroptosis, HO• attacks the PUFAs of membrane phospholipids. Phosphatidylethanolamine, which contains arachidonic (C_20_H_32_O_2_) or adrenic (C_22_H_36_O_2_) acids as a polyunsaturated fatty acid, is the substance that most often undergoes peroxidation. The products of membrane phospholipid peroxidation in a tumor cell accumulate not only due to the activation of the Fenton reaction, but also due to a reduced activity of the antioxidant defense system of the cell. The antioxidant defense system includes the selenoproteins GPX4 and GSH [19]. GPX4 uses GSH as an electron donor to reduce potentially harmful lipid hydroperoxides to non-toxic alcohols. Oxidized glutathione is reduced by the glutathione reductase that is constitutively activated in the cell [20]. Detoxification of lipid peroxides by GPX4 is limited by the presence of cystine, a GSH precursor, in the cell, which is transported via the xC-system [21]. In the cell, glutamate is exchanged for cystine in a 1:1 ratio. Cystine is reduced to cysteine by various reductases, in particular thioredoxin reductase 1, and is used as a building block for GSH biosynthesis. That the xC-system plays a key role in disease progression is confirmed by the results of clinical observations: the relapse rate of xC-positive tumors is significantly higher than that of xC-negative ones. GPX4 and the xC-system are considered potential targets for altering the redox status of the cell. Erastin, which blocks transport of cystine into the cell, remains the “gold standard” for ferroptosis induction. The second group of ferroptosis inducers includes GPX4 inhibitors (mainly RAS-selective lethal 3 (RSL3) and RASselective lethal 5 (RSL5)).

Phospholipid peroxidation disrupts protein–lipid interactions, alters the activity of membrane-bound enzymes, and affects membrane permeability. Continuous, intensive oxidation of membrane phospholipid PUFAs induces plasma membrane rupture and cell contents leakage into the intercellular environment. DAMPs in the tumor microenvironment (they may be divided into two subgroups, adjuvant and antigen) promote enhanced tumor infiltration by CD8^+^ T cells, maturation of dendritic cells, and increased phagocytic activity by macrophages [22, 23]. Therefore, ferroptosis in tumor cells is involved not only in the direct induction of cell death, but also in the reprogramming of the immune system, generating a specific immune response to tumor antigens, which should lead to the destruction of more tumor cells.

The oncosuppressive role of ferroptosis was first shown in triple-negative breast cancer [24]. The pronounced dependence of tumor growth on glutamine was indicative of a decrease in xC-system activity in the uptake of cystine. Further, sorafenib, a tyrosine kinase inhibitor, was found to deplete glutathione reserves in the cell, by blocking the xC-system, and trigger ferroptosis [25]. PARP inhibitors have exhibited similar action [26]. Ferroptosis in tumor cells is also induced by compounds that block GPX4 activity (e.g., altretamine, an FDA-approved alkylating agent) [27]. It is interesting to note that resistance to PD-1/PD-L1 immunotherapy is also believed to be associated with resistance to ferroptosis [28]. Even the clinical data mentioned in this brief review indicate that ferroptosis most likely contributes to the effects of antitumor drugs.

## MICROENVIRONMENT IN TUMOR PROGRESSION

Continuous growth of the tumor mass, when the tumor’s blood supply is inadequate, is accompanied by partial cell death. Dying cells induce the production of pro-inflammatory cytokines (TNF-α and -β, IL-1, IL-6, IFN-γ, etc.) [29]. The role of the cytokines is to regulate the body’s immune response to the inflammation caused by tissue damage. In response to the immunostimulatory signals released by dying cells, immunocompetent cells migrate to the tumor microenvironment and secrete pro-inflammatory cytokines. High concentrations of pro-inflammatory cytokines bring the tumor to a more aggressive growth phase [30]. As the tumor mass grows, the amount of dying tumor cells and the number of antigens in the tumor microenvironment increase: the inflammation becomes chronic. Chronic overexpression of pro-inflammatory mediators is observed at all stages of cancer development: inflammation severity is significantly higher in metastatic tumors than it is in the early stages of the disease [31].

Molecular changes initiated by tumor adaptation to a lack of nutrition, oxygen, and energy activate resident resting fibroblasts. Cancer-associated fibroblasts (CAFs) virtually rebuild the extracellular matrix by secreting vimentin, laminin, fibronectin, and collagen, the major scaffold protein of the extracellular matrix. Compaction of the extracellular matrix stimulates malignant tumor growth. The rigidity of the extracellular matrix acts as a barrier preventing drug penetration into the tumor (see review [32]).

Malnutrition during rapid tumor growth is accompanied by the formation of necrotic foci. DAMPs are released into the intercellular space, which leads to dendritic cell-mediated antigen uptake and presentation, as well as induction of a cytotoxic T cell response. As the tumor progresses, the reactive capabilities of T cells decreases. T cells switch to an anergy state that is characterized by decreased cytolytic activity and a reduced T cell proliferation index (see review [33]).

The immune response to DAMPs also involves macrophages (see review [34]). The cytotoxic activity of macrophages at the initial stages of tumor infiltration by macrophages retards tumor progression, but it is not enough to control tumor growth. The antitumor immune response is suppressed by the polarization of macrophages to the M2 phenotype. By secreting growth factors, cytokines, and extracellular matrix components, M2 macrophages enhance the malignant potential of tumor cells.

Tregs are the central link in the regulation of the immune response to both self- and tumor antigens. Normally, Tregs prevent the development of autoimmune diseases. The main function of Tregs in the tumor is to inhibit the proliferation of CD4^+^ and CD8^+^ T cells (see review [35]). It is very important that Tregs create an immunodeficient space in the tumor, which is suitable for bacterial growth. An increase in the number of Tregs in the tumor was shown to significantly raise prostate cancer mortality rates, regardless of other clinical factors [36].

The tumor recruits MDSCs from the blood to maintain immunosuppression. In malignancies, myeloid suppressors suppress the response of T and NK cells. MDSCs also express the CD40 that induces the accumulation of Tregs in the tumor microenvironment (see review [37]). It is interesting to note that Tregs, MDSCs, and M2 macrophages are resistant to ferroptosis, and that CD8^+^ T cells are sensitive to Fe-dependent death [38].

## PRO-INFLAMMATORY INTERLEUKINS IN THE TUMOR MICROENVIRONMENT

Interleukins, low-molecular weight proteins, are synthesized primarily by immune system cells and are divided into pro-inflammatory (IL-1, -6, -12, TNF-α, interferons, chemokines, IL-8, etc.) and anti-inflammatory (IL-4, -10, -13, and -17) (see review [39]).

IL-6 expression dominates in the tumor microenvironment (see review [40]). IL-6 levels are elevated in breast, cervical, colon, esophageal, head and neck, ovarian, pancreatic, and prostate cancers, as well as in patients with non-small cell lung cancer and multiple myeloma [41]. Abundant clinical data have been accumulated, confirming a correlation of IL-6 with resistance to therapy [42] and activation of metastasis (see review [43]). IL-6 binding to its receptor (IL-6Ra, gp80) and co-receptor, gp130, activates the JAK2/STAT3 signaling pathway [44]. STAT3 belongs to the family of pro-oncogenic transcription factors that are closely associated with inhibition of apoptosis, proliferation of tumor cells, and activation of metastasis and angiogenesis [45]. Hyperactivation of the IL-6/IL-6R/JAK2/STAT3 signaling pathway is observed in almost all types of tumors [46]. It is important to note that the level of IL-6 circulating in the blood of patients is a prognostic marker for both the disease course and the tumor response to therapy [47].

High concentrations of both IL-1α and IL-1β are found in the tumor microenvironment [48]. IL-1α and IL-1β levels are significantly increased in melanoma, colon, lung, and breast cancers, head and neck tumors and are associated with a tumor’s transition to the aggressive growth phase [49]. In genotoxic stress, increased production of IL-1α and IL-1β and their secretion activate tumor blood supply (see review [50]). There also exist data on a correlation between IL-1β expression and the formation of distant metastases [51]. Binding of IL-1α to its receptor activates expression of the pro-oncogenic transcription factor NF-kB that blocks Fasdependent apoptosis and provides conditions for tumor survival and progression [52]. It is becoming evident that high concentrations of pro-inflammatory cytokines in the tumor microenvironment are organic components of malignant tumor growth. Many recent studies have bolstered the idea that progression of malignancies is driven by smoldering inflammation.

## PRO-INFLAMMATORY CYTOKINES IN FERROPTOSIS IN CANCER

As noted above, the tumor reprograms the metabolism of iron and promotes its accumulation in the cell. It would seem that high iron concentrations inside the tumor cell should activate ferroptosis. However, ferroptosis is blocked in the tumor. In head and neck squamous cell carcinoma, IL-6 was shown to stimulate the expression of xC-system proteins [53]. Inhibition of the xC-system in these cells restored ferroptosis. Genetic knockdown of xC-system proteins reduced cell proliferation *in vitro*. Thus, it has been experimentally confirmed that the pro-inflammatory cytokine IL-6 blocks ferroptosis by activating the xC-system. Involvement of intratumoral immunosuppression in the blocking of ferroptosis has also been confirmed in subcutaneous tumor xenograft mouse models. RSL3, an inhibitor of GPX4, suppressed tumor growth in athymic nude mice [54]. Another study demonstrated the antitumor effect of imidazole ketonerastine (IKE), a ferroptosis inducer, in an immunodeficient mouse lymphoma model [55].

The induction of ferroptosis in tumor cells also involves other pro-inflammatory cytokines. IFN-γ in hepatocellular carcinoma cells was shown to block transcription of the *SLC7A11 *gene that encodes a subunit of the xC-system [56]. Under conditions of GSH deficiency, accumulation of phospholipid peroxidation products triggers ferroptosis. SLC7A11 and SLC3A2 expression in tumor cells was also suppressed by TNF-α: decreased cystine uptake led to cell death due to oxidative stress development [57]. It is important to note that IFN-γ- or TNF-α-induced ferroptosis can develop only at the initial stages of the disease; when the tumor switches to the aggressive growth phase, a shift towards IL-6 secretion occurs. In the aggressive growth phase, intratumoral IL-6 levels are manyfold higher than those of other cytokines: ferroptosis in cells with a highly malignant phenotype is blocked [58].

The next regulator of oncogenesis-associated inflammation is the transcription factor NF-kB that is activated in response to pro-inflammatory cytokines, the insulin-like growth factor, and the tumor necrosis factor. NF-kB is involved in the regulation of the cell cycle, proliferation, adhesion, and migration control, as well as in angiogenesis and invasion (see review [59]). There exists experimental evidence that NF-kB is also involved in ferroptosis. In U87 glioblastoma cells, RSL3, an inhibitor of GPX4, was shown to activate the NF-kB signaling pathway. Active NF-kB triggers ferroptosis by reducing the expression of SLC7A11, a subunit of the xC-system, and GPX4 [60]. As a result, lipid hydroperoxide concentrations increase and ferroptosis is triggered. In subcutaneous xenografts, inhibition of NF-kB by BAY 11-7082 abolished the antitumor effect of RSL3. Therefore, during tumor progression, the tumor develops mechanisms to avoid ferroptosis. Apparently, resistance to ferroptosis in a setting of high intracellular iron concentrations is another determinant that allows the tumor to escape antitumor therapy. The revealed resistance to ferroptosis, which is induced by the pro-inflammatory tumor microenvironment, not only expands our knowledge of the mechanisms underlying malignant disease progression, but also shifts the emphasis in interpreting the significance of intratumoral immunosuppression in carcinogenesis. Given the constitutive activity of the IL-6/JAK2/STAT3 signaling pathway in malignant diseases [61], resistance to ferroptosis may be considered a necessary condition for tumor progression.

## CONCLUSION

Although programs for the early detection of malignancies have significantly improved the chances of survival for cancer patients, drug resistance remains a serious impediment in cancer treatment. The realization that cells that survive chemo-, radio-, and targeted therapy are sensitive to ferroptosis has significantly increased interest in ferroptosis. The death of a therapy-resistant cell is induced by additional oxidative stress by Fenton reaction-generated hydroxyl radicals: the antioxidant defense system of the cell is almost completely destroyed. Strategies for using ferroptosis in the treatment of metastases open up new opportunities in cancer therapy. In preclinical models, ferroptosis inducers have caused relatively limited toxic effects in normal cells and demonstrated good tolerability. The randomized study Functional Assessment of Cancer Therapy–Lung Cancer in non-small cell lung cancer patients revealed that the humanized anti-IL-6 antibody (ALD518) delayed cachexia by reducing weight loss from 1.5 kg/month to 0.19 kg/month and increased the relapse-free survival time patients by 2.2 months [62]. ALD518 did not significantly affect tumor growth. Apparently, the use of anti-IL-6 antibodies is not enough to block tumor growth, although monotherapy improves the quality of life of patients. Of significant interest are the preliminary results of clinical studies on the combined use of ferroptosis inducers and antitumor drugs in ovarian cancer, triple-negative breast cancer, prostate cancer, colorectal cancer, and hepatocellular carcinoma (see review [63]). In addition, it may be hoped that the potential ability of ferroptosis to induce a specific immune response which enhances the therapeutic effect of other treatments (see review [64]) will prolong remission in cancer patients.

## Abbreviations

Tf: transferrin
TfR1: transferrin receptor 1
FPN: ferroportin
GPX4: glutathione peroxidase 4
DAMP: damage-associated molecular pattern
PARP: poly(ADP-ribose) polymerase
CAF: cancer- activated fibroblast
MDSC: myeloid-derived suppressor cell
FDA: Food and Drug Administration
IFN: interferon
Treg: regulatory T cell
TNF: tumor necrosis factor
IL-6: interleukin 6
GSH: glutathione
xC-system: cysteine/glutamate antiporter system
PUFA: polyunsaturated fatty acid
